# Influence of professional dental hygiene on oral and general health of retirement home residents: A comparative study

**DOI:** 10.1002/cre2.488

**Published:** 2021-09-01

**Authors:** Ingrid Peroz, Christoph Klein

**Affiliations:** ^1^ Department for Prosthodontics Charité – University Medicine of Berlin, Charité Centre for Dentistry, Gerodontology and Craniomandibular Disorders Berlin Germany; ^2^ Private Practice Berlin Germany

**Keywords:** geriatric patients, long‐term care facilities, professional oral hygiene

## Abstract

**Objectives:**

The oral status of nursing home residents is poor. This could compromise general health. The controlled study investigated the influence of quarterly professional dental hygiene interventions on oral and general health of elderly.

**Material and Methods:**

152 participants (mean age 84 years) of two residents' homes were examined. Parameters of general health, a questionnaire for caregivers, and oral parameters were evaluated at baseline and after 1 year. All caregivers were given one lesson on oral hygiene at baseline. In one home professional oral hygiene was performed every 3 months. Statistical analyses were done by Chi^2^ test for nominal data and t‐test for numeric data.

**Results:**

There were no significant differences between both homes regarding general health. Some oral parameters—if any—may be positively influenced by the intervention such as pocket depth, and Denture Hygiene Index and alterations of the mucosa.

**Conclusions:**

A quarterly professional hygiene is not able to influence general health and has—if any—little effect on oral health. This underlines the necessity for frequent interventions. An optimization of the health policy framework is necessary to allow caregivers more time for oral hygiene and to establish the accessibility of frequent professional health care for inhabitants in residents' homes.

## 1 INTRODUCTION

Demographic data describe an aging society nationally and internationally (Puturidze et al., 2018). An increasing number of elderly people are living in long‐term care facilities (LTCFs; Dahm et al., 2015). Examination of their oral health reveals a poor situation in terms of dental status, periodontal situation and cleanliness of the dentures, the tongue or the oral cavity (Al Baker et al., 2017; Bilder et al., 2014; Henriksen et al., 2004; Klotz et al., 2020; Nitschke & Muller, 2004; Ozkan et al., 2011; Simons et al., 2001; van der Putten et al., 2014; Weyant et al., 1993). This impaired oral health is influenced by multiple general health problems such as dementia, frailty, psychological disorders, or malnutrition and the according multiple drugs therapy (Klotz et al., 2020). A reduced oral health could influence the general health status, and especially regarding nutritional status, cardiovascular diseases, or aspiration pneumonia (Altenhoevel et al., 2012; Klotz et al., 2018; Komiyama et al., 2016; Paganini‐Hill et al., 2011; Rohrig et al., 2020).

Due to visual problems, reduced handgrip, sarcopenia, dementia, or financial problems, oral health care is neglected by elderly people. Additionally, the oral health of older aged people living in LTCFs is compromised due to limitations on the side of the caregivers: lack of knowledge about dental hygiene; lack of skills to deal with different kinds of dentures; poor attitude and low priority for oral health; time limitations; high turnover of personnel (MacEntee et al., 1999; Webb et al., 2015).

Interventions to improve this worsening situation have been made through the educational training of nurses and/or caregivers (Ho et al., 2019; Janssens et al., 2018). This may improve the sensitivity toward this issue. The inclusion of a dental hygienist in the organizing and education of oral health care in the LTCF seems to improve the oral hygiene (Amerine et al., 2014). Frequent and regular support of tooth brushing, denture cleaning, and professional dental care reduces the prevalence of aspiration pneumonia (Astvaldsdottir et al., 2018; van der Maarel‐Wierink et al., 2013), and is able to improve malnutrition and poor appetite (Astvaldsdottir et al., 2018).

In Germany, dental hygienists are allowed to treat patients only of prescribed and overseen by the dentist, not independently (BfJu, 1952). Twice a year the removal of calculus and plaque is covered by public health insurances in handicapped patients or in elderly people who need care. Further professional oral hygiene interventions have to be paid for privately by the patient.

Intervention studies, improving oral hygiene by regular tooth brushing, denture brushing or professional oral hygiene show less biofilm on dentures (Berteretche et al., 2012), significant improvement on the Oral Health Assessment Index, the Volpe‐Manhold Index, in nutritional status (Barbe et al., 2020), and a significant reduction in cases of aspiration pneumonia (van der Maarel‐Wierink et al., 2013). These improvements seem to depend on the frequency of the intervention, the cooperation of the health care providers and the kind of intervention (VRY et al., 2017).

The aim of the present controlled study was to investigate the influence of a quarterly professional dental hygiene treatment on general and oral health (Table 1). The frequency of intervention was chosen, as it seems realistic that two professional oral hygiene interventions would be covered by the health insurances per year and two could be paid by the patients themselves. According to the guideline for oral health care for institutionalized older people (De Visschere et al., 2011) an education for all caregivers was performed additionally by a dentist on oral and general health of inhabitants of LTCFs.

**Table 1:** List of parameters, evaluated in both examinations

	Outcome parameters
General health	BMI
	General diseases
	Medication
	Barthel‐Index
Questionnaire for care givers	
Dental health	DMFT
	Pocket depth
	Tooth mobility
	Approximal Plaque Index (API)
	Sulcus Bleeding Index (SBI)
	Denture Hygiene Index (DHI
	Alterations or oral mucosa

Abbreviation: BMI, body mass index.

## 2 MATERIAL AND METHODS

The study was performed in accordance with the Declaration of Helsinki and with the positive ethic vote of the Ethic‐Committee of the Charité (*EA2/033/08)*. All participants or their attendants gave their informed, written consent prior to their inclusion in the study.

**Figure 1:**
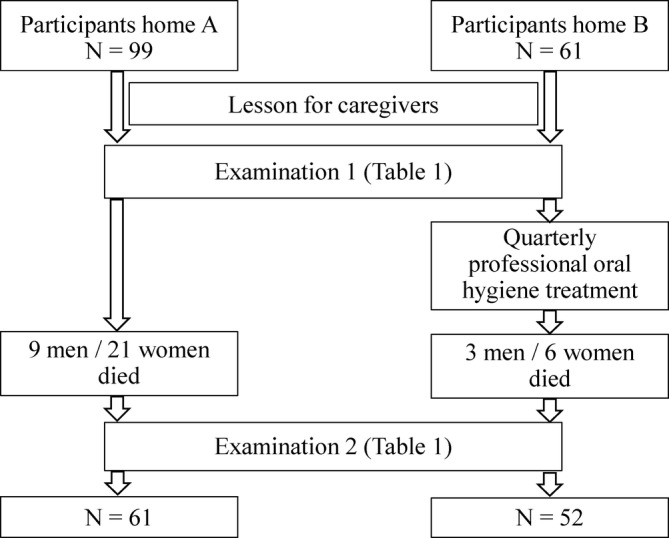
Flowchart of the comparative study

Two residents' homes took part in the study. In home A, 91 residents out of 110 gave their informed consent, in home B 61 of 99 residents agreed to take part. The health caregivers of both homes were trained with an oral presentation by a dentist. They got an e‐learning CD for further training on oral healthcare procedures in the elderly population. Parameters of oral and general health of residents were examined (Table 1). In residents home B, the participants additionally received a professional oral hygiene treatment every 3 months, depending on their dental status. After 1 year, all participants of the first examination were reexamined by the same dentist (Figure 1).

### 2.1 Examination of the participants

#### 2.1.1 General health

To evaluate the general health, beside age and sex the body mass index (BMI) was calculated as the quotient of weight divided by the body length^2^ (kg/m^2^). This was used to estimate the nutritional status: BMI <18.5 = underweight, BMI = 18.5–24.9 = normal weight, BMI = 25.0–29.9 = pre‐obesity, BMI = 30.0–34.0 = obesity class I, BMI = 35.0–39.9 = obesity class II, BMI > 40 = obesity class III.

The diseases and number of medications per day were acquired from the patients' records.

The Barthel Index was used to evaluate the functional ability of patients (Mahoney & Barthel, 1965). Patients' ability to perform 10 daily activities independently was evaluated (maximum 100 points).

#### 2.1.2 Oral health

The oral health parameters included the dental status, indicating the decayed (D), missed (M), and filled (F) teeth (T) as on the DMFT Index. The highest value is 28 as the wisdom teeth are not registered.

The pocket depth was evaluated mesially and distally on natural teeth. Decayed teeth were not included. The pocket depth was clustered in <4 mm, = 4 mm and >4 mm.

The Approximal Plaque Index (API) was used according to Lange (Lange et al., 1977). The API indicates the quality of the oral hygiene: API = 70–100% = insufficient oral hygiene; API = 35–70% = moderate oral hygiene; API = 25–35% = good oral hygiene; API ≤25% = excellent oral hygiene.

The Sulcus Bleeding Index was registered according to Lange (Lange et al., 1977). This can be used to grade the inflammation of the gingiva: SBI <10% = normal parodontium; SBI = 10–12% = mild inflammation; SBI = 21–50% = moderate inflammation; SBI >50% = severe and generalized inflammation.

The tooth mobility is graded in 1 = horizontal mobility less than 1 mm; 2 = horizontal mobility between 1 and 2 mm; 3 = horizontal mobility higher than 2 mm.

The Denture Hygiene Index (DHI) according to Wefers was used to estimate the cleanliness of the dentures (Wefers et al., 1991). The dentate surface of the dentures is divided into sextants in the oral and vestibular area. The basis of the denture on the mucosal side is divided into quadrants. It is registered whether these surfaces are clean (0) or contain plaque (Puturidze et al., 2018). The highest value is 10 for a denture with plaque on all surfaces.

#### 2.1.3 Questionnaire for caregivers

The caregivers were asked whether the participants perform their oral hygiene independently supported or if it is done completely by the nurses. Furthermore, the cooperativeness of the participants during oral hygiene procedures was asked about. The frequency of oral hygiene was differentiated as less than once a day, once a day, and more than once a day (Table 2).

**Table 2:** Questionnaire for caregivers

	Nominal categories		
Support during oral hygiene	Independent	Supported	By caregivers
Cooperative during oral hygiene	Cooperative		Not cooperative
Frequency of oral hygiene	Once a day	>Once a day	<Once a day

#### 2.1.4 Professional oral hygiene of the participants

According to the dental status, the procedures differed:

1. In edentulous participant: Plaque and calculus were removed from the dentures by hand brushing and by ultrasonic bath for 20 min. The oral cavity was rinsed with chlorhexidine (CHX) 0.2% for 30 s. In patients with dysphagia, the oral cavity was wiped out with a swab, which was impregnated with CHX 0.2% solution.
2. In patients with natural teeth: The teeth were cleaned of plaque and calculus by scalers and Gracey curettes. The interdental spaces were cleaned by dental floss or interdental brushes. The surfaces beneath bridges or bars were cleaned with Superfloss (Procter & Gamble GmbH, Sulzbacher Str. 40, 65,823 Schwalbach/Ts. Germany). The teeth were polished with polishing brushes and polishing paste and fluorized with Elmex Gelée (CP GABA GmbH, Beim Strohhause17, 20,097 Hamburg, Germany).
3. Patients with natural teeth and dentures: Procedures (a) and (b) were performed.
4. Edentulous patients without dentures: the oral cavity was rinsed with CHX 0.2% for 30 s or moistened with a swap soaked in CHX 0.2%.

#### 2.1.5 Statistics

Statistics were done by SPSS version 23 (IBM Corporation, New Orchand Road, Armonk, NY). For the comparison of baseline characteristics of participants followed up with those lost to follow up and characteristics in home A and B Chi^2^ test was used for nominal data and t‐test for independent samples for numeric data. For the comparison of characteristics between baseline and follow‐up examination (E1 and E2) Chi^2^ test was used for nominal data and *t*‐test for dependent samples for numeric data. *p* Values <0.05 indicate significant differences.

## 3 RESULTS

Thirtynine persons were lost for follow up as they died. The comparison of the baseline characteristics of those followed up and those who were lost to follow up showed significant differences in three variables (Table 3).

**Table 3:** Comparison of baseline statistics of participants followed up and those lost to follow up

Characteristics	Followed up	Lost to follow up	*p*‐Value
Age (*A*/*SD*)	82.7 (10.4)	86.7 (8.2)	0.309
Sex			
Female	88/76%	28/72%	0.593
Male	25/69.4%	11/30.6%	
BMI (kg/m^2^) (*A*/*SD*)	25.9/4.5	24.4/4.2	0.082
Cardiovascular disease (*N*/%)	72/63.7%	22/56.4%	0.418
Dementia (*N*/%)	62/54.9%	22/56.4%	0.867
Depression (*N*/%)	27/23.9%	3/7.7%	**0.028**
Diabetes (*N*/%)	33/29.2%	11/28.2%	0.906
Pulmonary disease (*N*/%)	16/14.2%	7/17.98%	0.569
Gastrointestinal disease (*N*/%)	26/23%	7% 17.9%	0.509
Urogenital disease (*N*/%)	33/29.2%	10/25.6	0.670
Musculo‐skeletal disease (*N*/%)	58/51.3%	16/41%	0.267
Morbus Parkinson (*N*/%)	14/12.4.3%	7/17.9%	0.386
Number of medication/d (*A*/*SD*)	8.2 (4.4)	6.4 (3.2)	**0.015**
Barthel‐index (*A*/*SD*)	55.7 (30.2)	44.4 (33)	0.921
Edentulous (*N*/%)	53/46.9%	22/56.4%	**0.031**
DMFT‐Index (*SD*)	25.6 (5.2)	26.2 (4)	0.280
Pocket depth >4 mm (% of teeth/*SD*)	6.0 (7.1)	6.1 (5.1)	0.526
API	88.2 (21)	89.5 (26.5)	0.899
SBI	91.8 (15.9)	90.2 (26)	0.324
Tooth mobility 1 (% of teeth)	1/1.8%	3/4–2%	Number too low
Tooth mobility 2 (% of teeth)	3/1.8%	1/5.9%	Number too low
DHI/OK (*A*/SD)	6.6 (3.4)	6.6 (3)	0.191
DHI/UK (*A*/SD)	6 (3.5)	5.7 (3.2)	0.407
Papillary hyperplasia (*N*/%)	2 /1.8%	0	0.403
Rhagades (*N*/%)	59/52.2%	19/48.7%	0.707
Inflammation (*N*/%)	70/61.9%	21/53.8%	0.374

*Note*: *N*, number of patients. A, average; *SD*, standard deviation.

Table 4 shows all examined characteristics of persons in home A and B at baseline (E1) and 1 year later (E2). There were no statistical differences between the residents of homes A and B at baseline and re‐evaluation after 1 year due to age, gender, and BMI. The BMI indicates a pre‐obesity with 25.6 ± 4.8 kg/m^2^ over all participants (Table 4).

**Table 4:** General health and oral health parameters in residents' home A and B at first examination (E1) and re‐examination after 1 year (E2)

Parameter	Home A at E1	Home B at E1	*p* Value A vs. B/E1	Home A at E2	Home B at E2	*p* Value A vs. B/E2	*p* Value Home A E1 vs. E2	*p* Value Home B E1 vs. E2
Participants (*N*)	91	61	n.s.	61	52	n.s.	n.s.	n.s.
Men/women (*N*)	26/65	10/51	n.s.	17/44	7/45	n.s.	n.s.	n.s.
Age (*A*/*SD*)	82.7/11.2	85.3/7.7	n.s.	81.2/11.7	86.4/7.8	n.s.	n.s.	n.s.
BMI (kg/m^2^/*SD*)	24.8/4.7	26.5/4	**0.02**	24.2/5.2	27.6/4.6	n.s.	n.s.	n.s.
Cardiovascular disease	54%	73%	**0.013**	61%	75%	n.s.	n.s.	n.s.
Dementia	58%	51%	n.s.	77%	50%	**0.003**	n.s.	n.s.
Depression	14%	28%	**0.039**	18%	19%	n.s.	n.s.	n.s.
Diabetes	30%	28%	n.s.	33%	25%	n.s.	n.s.	n.s.
Pulmonary Disease	12%	20%	n.s.	16%	19%	n.s.	n.s.	n.s.
Gastrointestinal disease	22%	21%	n.s.	26%	23%	n.s.	n.s.	n.s.
Urogenital disease	24%	34%	n.s.	34%	31%	n.s.	n.s.	n.s.
Musculo‐skeletal disease	42%	59%	**0.037**	48%	71%	**0.011**	n.s.	n.s.
Morbus Parkinson	16%	10%	n.s.	18%	10%	n.s.	n.s.	n.s.
Number of different medication (N/d/*SD*)	5.6/2.7	6.5/2.7	n.s.	5.5/3.2	6.5/3.1	n.s.	n.s.	n.s.
Barthel‐Index	52.9	52.7	n.s.	42.5	46.4	n.s.	**0.048**	n.s.
edentulous	54%	43%	n.s.	52%	38%	n.s.	n.s.	n.s.
DMFT‐Index/*SD*	26/4.2	25/5.8	n.s.	26/4.3	26/4.1	n.s.	n.s.	n.s.
Pocket depth >4 mm (% of teeth)	25.3%	30.5%	n.s.	40%	23%	n.s.	**0.014**	n.s.
API/*SD*	92.4%/19.8 5	84%/24%	**0.043**	90%/18.7%	88%/15%	n.s.	n.s.	n.s.
SBI/*SD*	95%/17%	87%/19.2%	**0.012**	97%/8.2%	80%/23.3%	n.s.	n.s.	n.s.
Tooth mobility 1 (% of teeth)	6.8%	7.7%	n.s.	24.5%	10.7%	**0.021**	**0.000**	n.s.
2 (% of teeth)	4.4%	0.5%%	n.s.	2.1%	1.3%0	n.s	n.s.	n.s.
DHI/UD (*A*/*SD*)	6.1/3.2	7.4/3.3	**0.041**	6.9/3.1	6.0/3.4	n.s.	n.s.	n.s.
DHI/LD (*A*/*SD*)	4.8/3	7.2/3.4	**0.001**	6.5/3.1	5.7/3.3	n.s.	**0.02**	**0.042**
Papillary hyperplasia (*N*)	1%	2%	n.s.	8%	0%	**0.035**	**0.028**	n.s.
Rhagades (*N*)	47%	57%	n.s.	79%	69%	n.s.	**0.020**	n.s.
Inflammation (*N*)	53%	70%	**0.029**	57%	50%	n.s.	n.s.	**0.026**

*Note*: *p*‐Values are given for the comparison of home A versus home B (A vs. B) and the time dependent comparison auf home A or B at E1 versus E2 (E1 vs. E2).

Abbreviations: *A*, average; *SD*, standard deviation; DHI, Denture Hygiene Index; LD, lower denture; UD, upper denture.

### 3.1 General health

In home B, more inhabitants suffered from cardiovascular diseases, depression, and musculoskeletal diseases at baseline. Only musculoskeletal diseases were more frequent in home B at re‐evaluation. There were changes over time for frequent general diseases. In home A, significantly more participants had dementia after 1 year.

According to the Barthel‐Index, more than half of the participants in both LTCFs need support. The Barthel‐Index increased after 1 year in home A only.

### 3.2 Oral health

Edentulism and DMFT did not differ between the two LTCFs or over time.

The pocket depth was evaluated mesially and distally for 338 teeth in home A and 428 teeth in home B. Decayed teeth were not included. Only the group of teeth with >4 mm pocket depth showed significant reduction in the intervention at the LTCF home B during the observation period.

The API and SBI were worse in home A at baseline examination. This difference diminished after 1 year.

Tooth mobility grade II was significant more often in home A than in home B at baseline evaluation and was reduced 1 year later in home A.

The cleanliness of the upper dentures was worse in home B at baseline, but equal in both homes after 1 year. The improvement of the DHI—upper denture was not significant. The DHI of the lower dentures was also worse in home B but improved over the observation period in home B and worsened in home A.

The number of patients with rhagades of the corner of the mouth and papillary hyperplasia of the palate did not differ at baseline examination but increased in home A, whereas the papillary hyperplasia reduced in home B.

Signs of inflammation of the mucosa were significantly more often in intervention home B at baseline and improved over 1 year in the same home.

### 3.3 Questionnaire for caregivers

The data of the questionnaire for the caregivers are given in Table 5. Both homes did not differ significantly at baseline or at follow up examination due to support during oral hygiene. The percentage of participants who were able to carry out their oral hygiene independently was reduced in both homes after 1 year. The reduction was significant in home B only (*p* = 0.049).

**Table 5:** Comparison of questionnaires for caregivers in home A and B during baseline examination (E1) and 1 year later (E2)

		Home A at E1 (%)	Home B at E1 (%)	*p* Value A vs. B/E1	Home A at E2	Home B at E2	*p* Value A vs. B/E2	*p* Value Home A E1 vs. E2	*p* Value Home B E1 vs. E2
Support during oral hygiene	Independent	38	56	n.s.	30%	44%	n.s.	n.s.	**0.049**
	Supported	38	31		40%	23%			
	By caregivers	24	13		30%	33%			
Cooperative during oral hygiene	Cooperative	81	93	**0.028**	77%	92%	n.s.	n.s.	n.s.
	Non cooperative	19	7		23%	8%			
Frequency of oral hygiene	Once a day	8	5	**0.000**	2%	4%	**0.001**	n.s.	n.s.
	>Once a day	71	95		66%	92%			
	<Once a day	21	0		32%	4%			

Most of the participants were cooperative during oral hygiene procedures In home B at baseline and at follow‐up examination, more participants were cooperative during oral hygiene. There was no time dependent effect.

The frequency of oral hygiene was higher in home B at baseline and at follow up examination‐ In home A, the frequency of oral hygiene showed the tendency to deteriorate, however without significance.

## 4 DISCUSSION

### 4.1 Study‐design

The prospective controlled study design included two LTCFs. Due to ethical considerations, the caregivers of both homes were instructed on the importance of oral health and the possibilities of oral hygiene procedures. By the quarterly interventions of the dentist, the caregivers of home B were reminded regularly on the importance of oral hygiene. This could have biased the improvement in the oral hygiene of the intervention group (Zenthöfer et al., 2013).

The examinations of all participants were performed in the residents' homes. The participants were seated on normal chairs or lying in bed. The dentist wore a head lamp for illumination of the oral cavity. Nevertheless, lighting conditions were not optimal so that measuring of pocket depth or examination of caries was compromised. A calibration of the dentist was not necessary, as the outcome parameters are routinely evaluated by dentists. However, the dentist was not blinded, therefore, a bias is possible.

Both LTCFs differed in the number of residents but were equal when comparing care standards. The average age of the participants is comparable to LTCFs in Europe (Jager et al., 2009; Janssens et al., 2017). The drop outs differ in three characteristics from the participants followed up. The prevalence of depression and number of medication was higher in the follow up participants. These facts however would compromise the follow up group. Due to dental characteristics, the number of edentulous participants was higher in the drop outs. This fact strengthens the effects due to parodontal parameters like pocket depth but weakens the effects of denture hygiene and mucosal alterations, which are often induced by dentures.

The participants of both homes differed at baseline due to BMI, depression, and musculo skeletal diseases, which could be a further bias.

Interestingly significantly (*p* = 0.012) more participants died in home A (*N* = 30, 33%) than in home B (*N* = 9; 15%) over the observation period. The mortality rate in home A was higher than reported in other studies (Chen et al., 2010; Goldberg & Botero, 2008). This fact could not be explained by general health diseases, as in home B significantly more participants suffered from cardio‐vascular diseases. This difference may be mainly influenced by the location of the residents' homes. Home B is located near to a hospital, so that emergency patients could be treated in the quickest time, with a positive effect on survivability. Another cause could be the higher percentage of women in home B. Women generally has a higher life expectancy. The higher BMI in home B could also have a positive effect in the lower mortality there (Volkert et al., 1992).

The strength of the study is the long observation period of 1 year and the inclusion of parameters describing general health. Barbe et al. evaluated oral parameters after 3 months of professional tooth brushing every 3 weeks (Barbe et al., 2020). Amerine et al. evaluated the permanent support of a dental hygienist for 8 weeks on oral hygiene (Amerine et al., 2014). Morino et al. performed a professional oral care once a week for 1 month (Morino et al., 2014). Zenthöfer et al. observed oral parameters after instructional interventions with remotivation of participants or nursing personal after 3 months (Zenthöfer et al., 2013).

### 4.2 General health

The Barthel‐Index increased significantly in home A and was stable in home B. This do not reflect the intervention but more likely the development of dementia in both homes. Dementia is combined with the loss of ability to perform daily life activities like oral hygiene (Jockusch et al., 2020) and a cause of death in the elderly population (Goldberg & Botero, 2008).

The number of medication is lower than in the study by Rohrig et al. and similar to the study of Peltola et al. (2005); Rohrig et al. (2020) examined patients of a hospital, whereas Peltola et al. examined inhabitant of LTCFs. Analyzing the different medication groups, 85% of all participants took cardio‐vascular medication, followed by psychotropic drugs for 68% at baseline. Polypharmacy, especially the intake of antidepressants may cause xerostomia (Rohrig et al., 2020). Xerostomia is combined with more plaque retention and dysphagia and a reduced Oral Health Assessment Instrument (Rohrig et al., 2020).

Systematic literature reviews show slight influences in general health with the improvement of oral health (Astvaldsdottir et al., 2018; Rohrig et al., 2020; van der Maarel‐Wierink et al., 2013). The reduction of microbes through oral hygiene is able to reduce the prevalence of pneumonia in frail patients (Astvaldsdottir et al., 2018). However, the frequency of oral hygiene procedures was high and motivated: tooth brushing after every meal, professional oral hygiene once a week and denture cleaning once a day. Comparing this strategy with the performed oral hygiene in both homes of the present study, we have to admit that the reality is far away from the ideal standard.

The improvement in appetite and reduction in malnutrition also show possible associations with oral health (Astvaldsdottir et al., 2018). The present study failed to show any influence on BMI as a nutritional parameter.

### 4.3 Oral health

In concordance with other national and international studies, the participants of LCTFs showed a compromised dental situation with a high percentage of decayed, missing, or filled teeth and a strong need for dental treatment (Henriksen et al., 2004; Nitschke & Muller, 2004; Simons et al., 2001).

Other studies with more frequent interventions but shorter observation periods show significant improvements of oral hygiene and oral health (Amerine et al., 2014; Barbe et al., 2020; Morino et al., 2014; Zenthöfer et al., 2013). However, the study results of Barbe et al. evaluating professional tooth brushing every 3 weeks for 3 months showed no improvement of gingivitis or the presence of plaque (Barbe et al., 2020). The same could be seen in the present study. The improvements of pocket depth in home B could be a result of the intervention. But due to so many confounders as mentioned above, there arise some doubt. A reason for the improvement of denture hygiene in home B could be the higher percentage of women, who are regularly better in performing oral hygiene than men and the higher loss of edentulous participants due to death.

Improvements in oral mucosa alterations seem to underline the positive effect of professional oral hygiene on the oral health of the participants. According to the study by Klotz et al., no intervention to improve oral health results in worse conditions of the oral status (Klotz et al., 2020) as could be seen in home B. However, more edentulous participants were lost than dentate ones, a confounder which should be recognized.

### 4.4 Role of caregivers

The poorer motivation of the caregivers in home A with regard to cleaning the oral cavity did not improve over the observation time. It is much easier to clean dentures extra orally than to clean the teeth in the oral cavity. The cooperation of the patients also has to be taken into account. Home A had significantly more participants with dementia at the second evaluation and the percentage of noncooperative patients increased.

As all caregivers got an educational instruction, its impact on the oral hygiene could not be estimated. Nevertheless, other studies and reviews doubt, whether a single lesson would be helpful (Astvaldsdottir et al., 2018; De Visschere et al., 2012; Gammack & Pulisetty, 2009; Sjogren et al., 2010). Regular educational instructions of nurses (Janssens et al., 2018) and regular professional oral hygiene however are able to improve oral health (Amerine et al., 2014; Astvaldsdottir et al., 2018; Barbe et al., 2020; Komiyama et al., 2016; van der Maarel‐Wierink et al., 2013).

## 5 CONCLUSIONS

Within the limitations of this study we may conclude, that the intervention of a professional oral hygiene treatment every 3 months had no influence on the general health of the participants and—if any—very little on oral health like DHI, pocket depth, and oral mucosal alterations. The frequency of quarterly professional oral hygiene treatment seems realistic but not frequent enough to produce convincing positive effects on oral and general health. This underlines the necessity for daily oral hygiene procedures. Not only are instructions to caregivers necessary but also an improvement in the health policy framework to allow, for instance, oral hygiene in an adequate time span, regular professional health care by dental hygienists and the coverage of costs by health insurances.

## CONFLICT OF INTEREST

The authors declare no conflict of interest.

## AUTHOR CONTRIBUTIONS

Ingrid Peroz, responsible for the methodology, ethic approval, supervision, preparing of the manuscript. Christoph Klein, responsible for the acquisition of patients, execution of the study, data collection and statistical evaluation, preparing the manuscript.

## Data Availability

Data available on request from the authors The data that support the findings of this study are available from the corresponding author upon reasonable request.
